# IDO1 Inhibition Reduces Immune Cell Exclusion Through Inducing Cell Migration While PD-1 Blockage Increases IL-6 and -8 Secretion From T Cells in Head and Neck Cancer

**DOI:** 10.3389/fimmu.2022.812822

**Published:** 2022-03-14

**Authors:** Meri Sieviläinen, Jordan Saavalainen, Shady Adnan-Awad, Tuula Salo, Ahmed Al-Samadi

**Affiliations:** ^1^ Department of Oral and Maxillofacial Diseases, Clinicum, University of Helsinki, Helsinki, Finland; ^2^ Translational Immunology Research Program, Faculty of Medicine, University of Helsinki, Helsinki, Finland; ^3^ School of Biological Sciences, University of Edinburgh, Edinburgh, United Kingdom; ^4^ Clinical Pathology Department, National Cancer Institute, Cairo University, Cairo, Egypt; ^5^ Hematology Research Unit, Department of Hematology, University of Helsinki and Helsinki University Central Hospital Comprehensive Cancer Center, Helsinki, Finland; ^6^ Cancer and Translational Medicine Research Unit, University of Oulu, Oulu, Finland; ^7^ Medical Research Center, Oulu University Hospital, Oulu, Finland; ^8^ Department of Pathology, Helsinki University Hospital (HUS), Helsinki, Finland

**Keywords:** head and neck squamous cell carcinoma, microfluidic chip, immune checkpoint inhibitors, immunotherapy, PD-1, IDO1, PD-L1

## Abstract

**Background:**

Immune checkpoint inhibitors (ICIs), primarily anti-PD-1, are currently used to treat patients with recurrent or metastatic head and neck squamous cell carcinoma (HNSCC). However, only a minority of patients benefit from these costly therapies. Therefore, there is an unmet need to better understand the effect of ICIs on immune effector cells. This study aimed to investigate the effect of a PD-1 antibody and an IDO1 inhibitor on different lymphocyte populations (NK, CD4^+^, and CD8^+^ T cells) in term of migration, cytotoxicity, and cytokine release in the presence of HNSCC cells.

**Methods:**

Using a microfluidic chip, we injected HSC-3 cells (an oral tongue squamous cell carcinoma cell line) embedded in a human tumor-derived matrix “myogel/fibrin” together with NK, CD4^+^, and CD8^+^ T cells in separate channels. The two channels were connected with microchannels. The PD-1 antibody nivolumab and IDO1 inhibitor epacadostat were added to the microfluidic chips. Lymphocyte migration and cytotoxicity were examined under fluorescent microscopy and cytokine release was measured using a FirePlex Human Discovery Cytokines Immunoassay.

**Results:**

Epacadostat significantly increased the migration and infiltration of NK and CD4^+^ T cells, but not CD8^+^ T cells, towards the cancer cells. Nivolumab did not exhibit a similar effect. While CD8^+^ T cells alone showed near to no migration, adding CD4^+^ T cells enhanced migration towards the cancer cells. There was a mild nonsignificant increase in apoptosis of HSC-3 cells after adding epacadostat to lymphocytes. In contrast, HSC-3 proliferation was not affected by lymphocytes regardless of ICIs. Nivolumab significantly increased release of MIP1-α, IL-6, and IL-8 from NK, CD4^+^, and CD8^+^ T cells, respectively.

**Conclusions:**

This study revealed that each subpopulation of lymphocytes respond differently to ICIs. We also revealed the subpopulation of lymphocytes responsible for the increases in specific serum cytokines after ICI treatment.

## Introduction

Head and neck squamous cell carcinoma (HNSCC) is the eighth most common cancer worldwide and accounts for 3% of cancer-related deaths (1, 2). The incidence of HNSCC arising from the tongue and oropharynx has increased approximately 30% in the last 30 years (2). The increase in oropharyngeal squamous cell carcinoma incidence is related to the rise in human papillomavirus (HPV) infections; however, there is no known specific etiology for the growing incidence of HPV-negative tongue cancers (2, 3). Most HNSCCs are characterized by rapid metastasis and a high recurrence rate (4, 5). Currently, primary treatment of HNSCC patients consists of surgery and (chemo-)radiotherapy either alone or in combination (6). Immunotherapy is the newest treatment modality for recurrent or metastatic HNSCC (7, 8).

There are currently two immune checkpoint inhibitors (ICIs) approved for treating HNSCC: nivolumab (Opdivo^®^) and pembrolizumab (Keytruda^®^), both of which are humanized IgG4κ monoclonal PD-1 antibodies (9). PD-1 is an inhibitory receptor expressed on T cell surfaces. When bound to its ligand PD-L1 or PD-L2, PD-1 transduces a signal to the T cell resulting in its deactivation and inhibition of proliferation (10). Tumor cells utilize this mechanism that safeguards natural homeostasis to mediate immune escape *via* expressing a high abundance of PD-L1 on their membranes (10).

Despite being approved for clinical use, only a minority of HPV-negative HNSCC patients benefit from PD-1/PD-L1 axis-based drugs. Thus, other immunotherapies that can modify the immune landscape of the tumor to enhance the effect of existing ICIs should be identified (11–13). A promising target molecule may be indoleamine 2,3-dioxygenase 1 (IDO1), a cytosolic heme-containing enzyme that catalyzes the initial step of tryptophan catabolism kynurenine and its other immunosuppressive catabolites (14). At present, the two most advanced and investigated drugs for IDO1 inhibition are indoximod and epacadostat (13).

The research focus on ICIs is shifting towards addressing the challenges with the approved drugs. More in-depth knowledge is needed on the immune responses elicited by ICIs in the tumor microenvironment (TME) and how these therapies affect different lymphocyte populations (8). Our previous study (15) introduced a novel, three-dimensional (3D) humanized microfluidic chip assay to test the effect of ICIs on lymphocyte migration and cytotoxicity towards cancer cells. The use of microfluidic chip in studying immune cell migration was previously tested and validated by comparing it with a mouse xenograft model by Lucarini et al., 2017 (16). Here, we sought to explore the effect of ICIs nivolumab and epacadostat on the behavior of natural killer (NK), CD4^+^, and CD8^+^ T cells in the presence of HPV-negative HNSCC cells.

## Materials and Methods

### Cell Culture of Human HNSCC Cells

A HPV-negative, highly aggressive metastatic human tongue squamous cell carcinoma cell line (HSC-3, Japan Health Sciences Foundation, Japan) was cultured with Dulbecco’s Modified Eagle’s Medium (DMEM)/F-12 (Gibco Paisley, UK) supplemented with 10% heat-inactivated fetal bovine serum (FBS; Gibco), 100 µg/ml streptomycin (Gibco), 100 U/ml penicillin (Gibco), 5 µg/ml amphotericin b (Sigma-Aldrich, St. Louis, Missouri, USA), and 50 µg/ml ascorbic acid (PanReac AppliChem, Darmstadt, Germany) in 75 m^2^ flasks. Cells were divided after reaching 80% confluence with 10% trypsin/EDTA (Gibco). The HSC-3 cells were tested negative for mycoplasma using a PromoKine PCR Mycoplasma Test Kit I/C (PromoCell, Heidelberg, Germany). The cell line was authenticated by FIMM Technology Center (Helsinki, Finland).

### Lymphocyte Isolation

We obtained buffy coats from 6 healthy donors (median age 53 years, **Supplement Table 1**) provided by the Finnish Red Cross. The ethical committee of the Finnish Red Cross approved the sample collection (permission number 42/2020). First, we diluted the buffy coats 1:2 with sterile phosphate-buffered saline (PBS) without calcium and magnesium (Corning, Corning, NY, USA). Using a density gradient technique, we then isolated the peripheral blood mononuclear cells (PMNCs) with Ficoll-Paque™ Premium (Sigma-Aldrich) by centrifugation at 800 RPM, acceleration 1, and deceleration 0 for 30 minutes. The leukocyte ring was then collected in a falcon tube and washed twice with PBS. We utilized a MACS system with negative selection (Miltenyi, Biotec, Germany) to isolate NK cells (NK Cell Isolation Kit human, Miltney), CD4^+^ T cells (CD4^+^ T Cell Isolation Kit human, Miltney), and CD8^+^ T cells (CD8^+^ T cell Isolation Kit human, Miltney) according to the manufacturer's protocol.

The purity of the isolated lymphocytes was confirmed with a panel of fluorescent antibodies and flow cytometry. This included antibodies for CD3-APC, CD56-PE-Cy7, CD4-PE, and CD8-FITC (BD Biosciences, USA). Subsets for NK cells were identified as CD3^-^CD56^+^CD8^-^CD4^-^, CD4^+^ T cells as CD3^+^CD56^-^CD4^+^CD8^-^, and CD8^+^ T cells as CD3^+^CD56^-^CD4^-^CD8^+^. The cells were calculated with FACS-Verse (BD Biosciences) and analyzed using BD FacSuite™ software. Purities >90% were accepted (**Supplement Figure 1**).

### Viability Assay

To test if the ICIs used in this study affect cancer cell viability, a 96-well plate was seeded with HSC-3 cells at a density of 1000 cells/well in 100 µL of complete medium and incubated overnight. The following day, four different concentrations of nivolumab (Opvido^®^, Selleckchem, Houston, Texas, USA; 0.0005, 0.005, 0.05, and 0.5 µM) and epacadostat (MedChem Express, Monmouth Junction, New Jersey, USA; 0.0013, 0.013, 0.13, and 1.3 µM) were added to the media and incubated for 3 days. The plate was then taken to room temperature for 15 minutes before starting the assay. 100 µL of CellTiter-Glo (Promega, Madison, Wisconsin, USA) was dispensed in each well. The plate was shaken with a plate shaker (Heidolph, Schwabach, Germany) for 5 minutes at 450 RPM and then rotated gently for 5 min at 1000 RPM with a plate spinner (Thermo Scientific, Waltham, Massachusetts, USA). Cell viability was measured with BMG Pheraster FS (BMG Labtech, Germany).

Neither nivolumab nor epacadostat affected HSC-3 cell viability at any used concentration (**Supplement Figures 2A, B**, p-value > 0.05). We decided to use 0.5 µM nivolumab and 1.3 µM epacadostat, as these concentrations correspond to those in patient serum at the standard clinical dose (17, 18).

### Microfluidic Chip Assay

**Figure 1:**
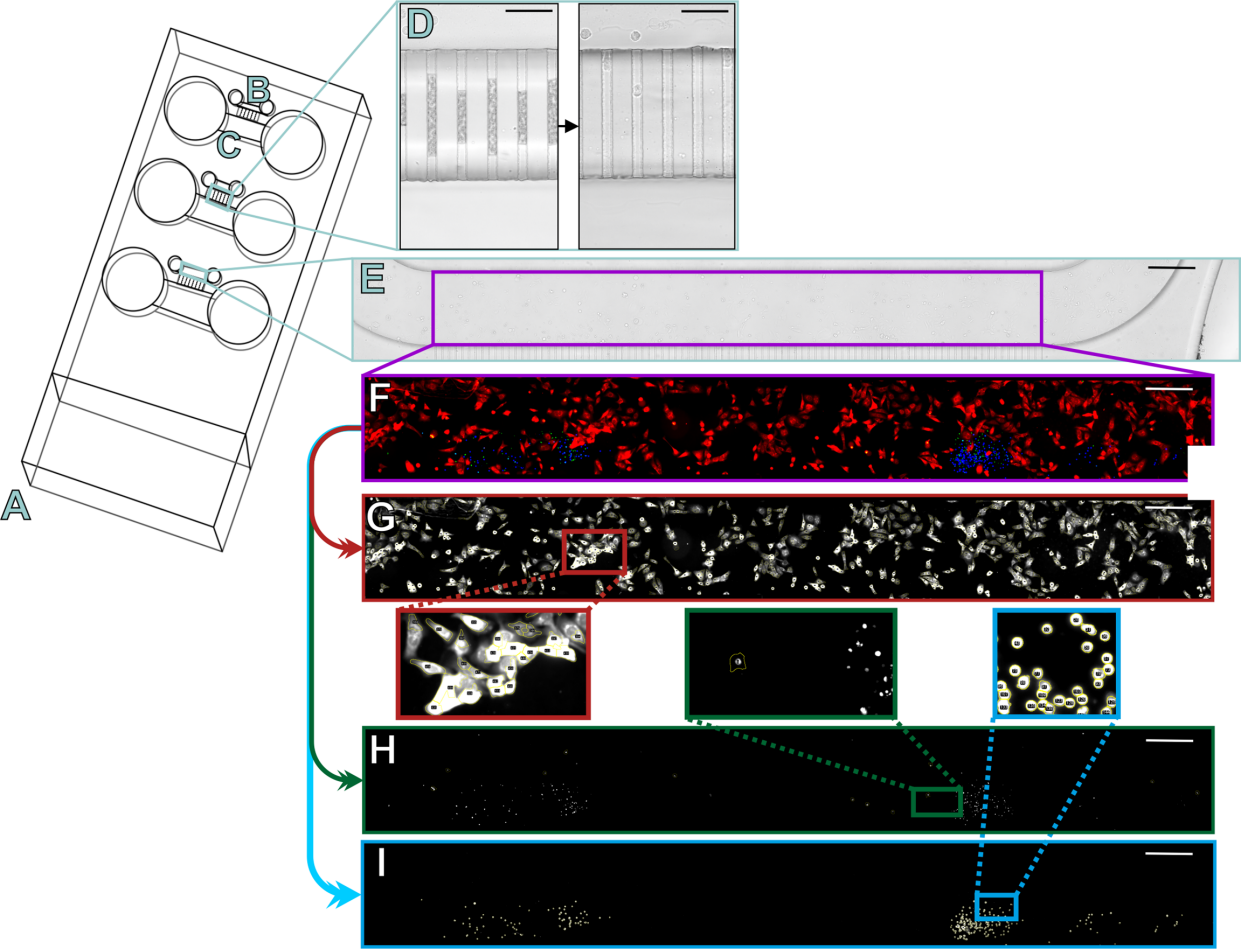
Schematic of the microfluidic chips and semi-automated analysis of the three-dye system. The slide design contains three microfluidic chips **(A)** into which cancer cells were injected into the smaller channels **(B)** and lymphocytes into the larger channels **(C)**. The two channels were connected by microchannels, which were initially filled by air but opened in the first 24 hours of injection **(D)**. The slides were imaged daily under a fluorescence microscope to obtain a multichannel image, which was then cropped for analysis to contain only the cancer cell channel (**E**; purple rectangle). The fluorescence signals were amplified and autofluorescence signals decreased manually with Image J to represent the channel **(F)** where cancer cells are shown in red, lymphocytes in blue, and apoptotic cells in green or yellow. Using the built-in features of ImageJ, our algorithm first separated the different channels for analysis and started by counting the positive cells for the red channel **(G)** based on a minimum threshold, shape, and size. The green channel **(H)** was counted by checking the red intensities for a minimum green intensity. The blue channel **(I)** was counted based on a minimum threshold, shape, and size. Scale bar **(D)** 50 µm and E-I 300 µm.

Our previously applied protocols (15) were adjusted for the Probiont™ microfluidic platforms. A schematic representation and phase-contrast microscopy images of the chip used in this work are shown in **Figure 1A**. The HSC-3 cells were stained with CellTrace™ Far Red (Invitrogen, Thermo Fisher) according to the manufacturer’s instructions. The cells were then suspended in Myogel/fibrin gel at the following concentrations: 2.4 mg/ml Myogel (lab-made; 19), 0.5 mg/ml fibrinogen (Merck, Darmstadt, Germany), 33.3 µg/ml aprotinin (Sigma-Aldrich), and 0.3 U/ml thrombin (Sigma-Aldrich) diluted in DMEM/F12 media with 10% of FBS. 5 µM of IncuCyte Caspase-3/7 Green (Sartorius, Göttingen, Germany) was added to detect apoptotic cells. The HSC-3 cells were divided into the following three groups: control without drug, 0.5 µM nivolumab, and 1.3 µM epacadostat. 2 µL of each cell suspension containing 1000 cells in gel were loaded into separate small “cancer cell channels” of the microfluidic chip (**Figure 1B**).

The lymphocytes (NK, CD4^+^, or CD8^+^ T cells) were stained with CellTrace™ Violet (Invitrogen) according to the manufacturer’s instructions. For coculture of CD4^+^ T cells and CD8^+^ T cells, CD4^+^ T cells were stained with CellTrace Violet and CD8^+^ T cells with CellTracker Orange (Invitrogen) according to the manufacturer’s instructions. Cell viability and number were measured using trypan blue solution utilizing CellCountess (Invitrogen). After staining, cells were suspended in DMEM/F12 media supplied with 10% FBS, 10 ng/ml recombinant human IL-2 (BioLegend, San Diego, California, USA; 20), and 5 µM Caspase-3/7 green (Sartorius). Lymphocytes were divided into the following groups: control without drug, 0.5 µM nivolumab, and 1.3 µM epacadostat. 100 µL of cell suspension, containing 100 000 viable lymphocytes, was added to the larger ‘lymphocyte channels’ of the chip (**Figure 1C**). In controls without lymphocytes, 100 µL of DMEM/F12 media supplied with 10% FBS and 5 µM Caspase-3/7 green were injected.

After injections, the chips were incubated for 72 hours in a cell culture laminar. The conditioned media was then collected from the chips and stored at -80°C until further analysis.

### Fluorescence Microscopy

Chips were imaged under a fluorescence microscope Nikon Ti-E with Alveole Primo (Nikon, Tokyo, Japan) connected to Hamamatsu Orca Flash 4.0 LT B&W camera (Hamamatsu Photonics, Hamamatsu, Japan) and Lumencor Sola SE II 365 (Lumencor, Beaverton, Oregon, USA). Images for the following transmitted light and fluorescent filters were acquired: DAPI (Semrock 5060C, excitation 377/50, emission 447/60), GFP (Semrock 3035D-NTE, excitation 472/30, emission 520/35), TxRed (Semrock 4040C, excitation 531/40, emission 593/40), and Cy5 (excitation 640/20, emission 700/75). Multiple chips were placed on a microscope slide adapter. The NIS-Elements Advanced Research program was automated to image at 20x magnification and to scan 10 images horizontally to form a complete representation of the channel producing a multichannel composite image. Images of the chips were acquired daily to confirm opening of the microchannels after 24 hours of incubation (**Figure 1D**).

### Semi-Automated Counting of Cells

Before analysis, multichannel images were cropped to contain just the cancer cell channel (**Figures 1E, F**). Using the built-in algorithms of Image J (NIH, National Institutes of Health, USA), we coded a semi-automated positive cell counter to quantify intensities in the three different channels of the composite image. The algorithm separated cancer cells from the background based on intensity and morphology (**Figure 1G**). All detected red cancer cells were analyzed for positive green intensity to calculate the apoptotic cancer cells with a minimum threshold to exclude artifacts (**Figure 1H**). The algorithm analyzed the number of lymphocytes from the blue channel based on intensity and morphology (**Figure 1I**).

The same counting method as in the three-dye system was utilized in the coculture chips of CD4^+^ and CD8^+^ T cells for the number of cancer cells, apoptotic cancer cells, and CD4^+^ T cells by making an individual scoring picture with only these three channels. To calculate the number of CD8^+^ T cells, the CD4^+^ T cell channel and green channel were removed and the CD8^+^ T cell orange channel was changed to blue to better visualize the results (**Supplement Figures 3A, B**). To differentiate between the background fluorescence from the red-stained cancer cells and the now blue CD8^+^ T cells, the algorithm calculated the number of any blue intensities with a positive red intensity (**Supplement Figure 3C**). Based on size and morphology, the algorithm then separately calculated the positive CD8^+^ T cells, from which the false-positive cell number of blue intensities with red was subtracted to gain the final number of CD8^+^ T cells (**Supplement Figure 3D**). To visualize the results clearly, we changed the cancer cells to greyscale and CD8^+^ T cells to magenta and added back the CD4^+^ T cell blue channel (**Supplement Figure 3E**).

The regions of interest (ROIs) were saved automatically and examined manually for any artifacts for all analyses.

### Cytokine Release

Conditioned media from the microfluidic chips were collected and diluted 1:1 with cell culture media. Cytokine profiling was performed by Abcam FirePlex Service (Boston, USA). Analysis was performed utilizing FirePlex^®^-96 Key Cytokines (Human) Immunoassay Panel (Abcam, Cambridge, UK), which detects the following 17 cytokines: granulocyte-macrophage colony-stimulating factor (GM-CSF), interleukin-(IL-)1beta, -2, -4, -5, -6, -8, -9, -10, -12p70, -13 and 17-A, IFN-γ, monocyte chemoattractant protein-1 (MCP-1), macrophage inflammatory protein 1 alpha (MIP1-α), macrophage inflammatory protein 1 beta (MIP1-β), and tumor necrosis factor-alpha (TNF-α). Each sample was analyzed in duplicate.

### Statistical Analysis

All experiments were repeated three times, each time using a different donor and each in duplicate. SPSS software program version 26.0 (IBM SPSS Statistics, SPSS, IL, USA) was utilized for statistical analyses. The proliferation rate of the cancer cells was calculated by dividing the number of cancer cells on days 2 and 3 by the number of cancer cells on day 1. One-way analysis of variance (ANOVA) followed by the Bonferroni *post-hoc* test was used to examine the statistical significance between the different groups.

Flow cytometer output for cytokine release was analyzed using FirePlex™ Analysis Workbench software (https://www.abcam.com/kits/fireplex-analysis-workbench-software). Concentrations were interpolated from the standard curve obtained in duplicate. Due to donor variation, the data were log-normalized and processed as fold changes (raw data included as **Supplement Table 2**). Statistical analysis for cytokine release was calculated with a One-Sample *t*-test.

P-values <0.05 were regarded as significant and are presented as follows: * <0.05 and ** < 0.01.

## Results

### Epacadostat, but Not Nivolumab, Increased NK and CD4^+^ T Cell Migration Towards HSC-3 Cells and Has a Mild Effect on HSC-3 Cell Apoptosis

**Figure 2:**
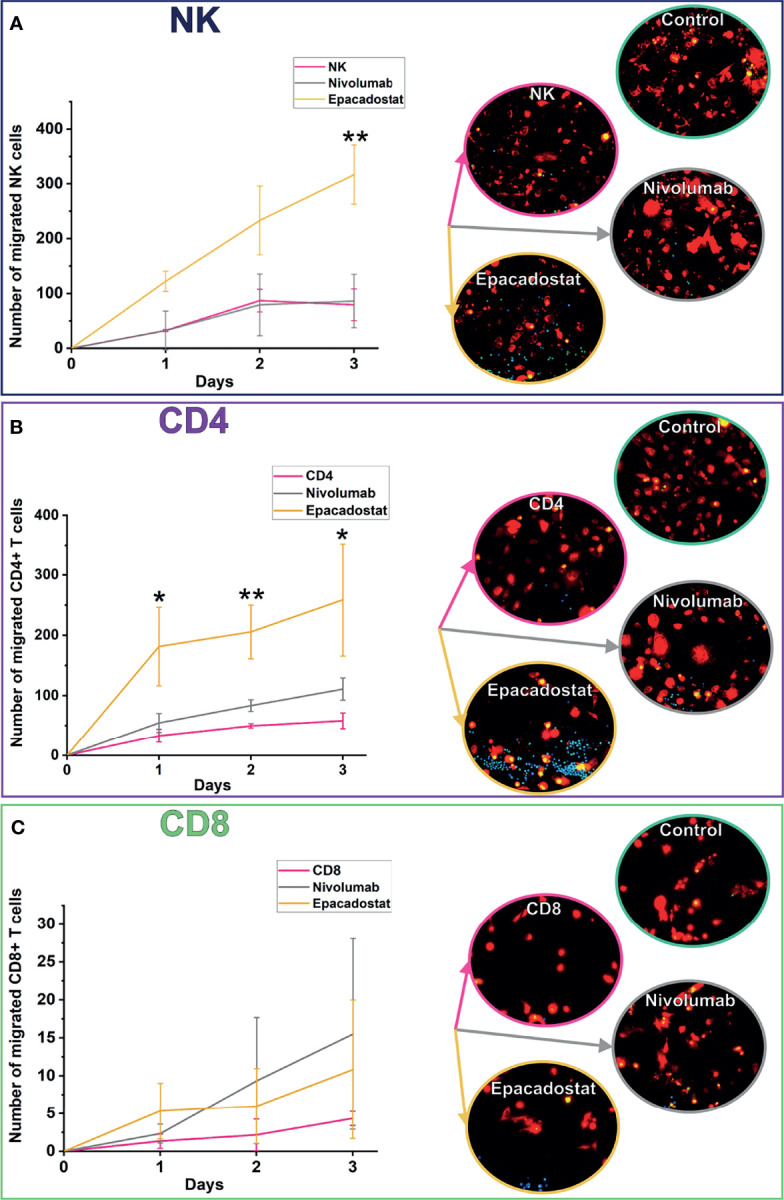
Migration of NK, CD4^+^, and CD8^+^ T cells towards cancer cells over 3 days. NK cells migrated significantly more with epacadostat incubation than in the NK control on day 3 (**A**; **p=0.009). The effect was observed also with CD4^+^ T cells but on all the 3 days of the experiment (**B**; day 1 *p=0.027, day 2 **p=0.003, day 3 *p=0.033). CD8^+^ T cells did not significantly differ in migration **(C)**. HSC-3 cells are shown in red, lymphocytes in blue, and apoptotic cells in green in the fluorescence images. Results are reported as the average number of lymphocytes migrated ± SD. The experiments were repeated three times, each time using a different donor and each in duplicate. *NK, natural killer cell; CD4, CD4^+^ T cells; CD8, CD8^+^ T cells*.

We previously reported that an IDO1 inhibitor enhances migration of PBMNCs towards carcinoma cells using HSC-3 cell line and two patients derived cancer cells (15). We investigated this migration further by studying specific subgroups of lymphocytes. Lymphocyte migration towards cancer cells was followed over 3 days. The IDO1 inhibitor epacadostat significantly induced both NK and CD4^+^ T cell migration compared to controls (**Figures 2A, B**). For NK cells, the effect was significant only by day 3 (p=0.009, **Figure 2A**). For CD4^+^ T cells, the effect was significant from the first day and over the 3 days (day 1 p=0.027, day 2 p=0.003, day 3 p=0.033, **Figure 2B**). On the other hand, the number of CD8^+^ T cells migrating towards cancer cells was minimal and was not affected by epacadostat stimulation (**Figure 2C**). As previously observed (15), PD-1/PD-L1 blockade did not affect migration of lymphocytes towards cancer cells. A full representation of channels is available as **Supplement Figures 4A–C**.

**Figure 3:**
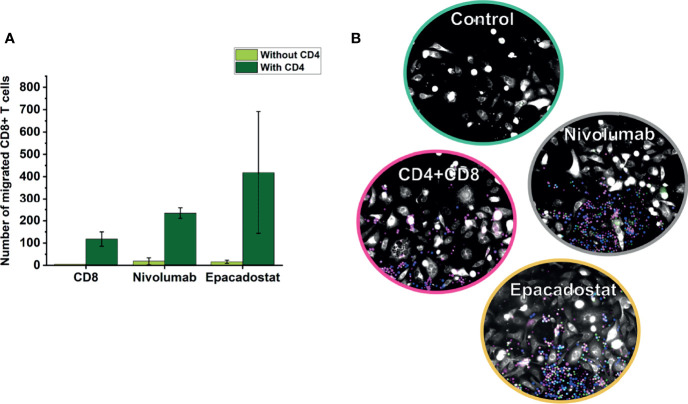
Migration of CD8^+^ T cells in coculture chips of CD4^+^ and CD8^+^ T cells over 3 days. CD8^+^ T cells overall migrated more in coculture with CD4^+^ T cells than alone **(A)**. In the representative image **(B)**, CD4^+^ T cells are shown in blue, CD8^+^ T cells in magenta, cancer cells in white, and apoptotic cells in green. Results are reported as the average number of lymphocytes migrated ± SD. The experiments were repeated two times, each time using a different donor and each in duplicate. Full channel representations are provided as **Supplement Figure 4**. *CD8=CD8^+^ T cells, CD4=CD4^+^ T cells*.

To study if adding CD4^+^ T cells could enhance CD8^+^ T cell migration, we cocultured these lymphocytes isolated from two donors. Coculturing CD4^+^ T cells with CD8^+^ T cells increased migration of CD8^+^ T cells towards the cancer cells approximately ten-fold when compared with CD8^+^ T cell monoculture (**Figure 3**). However, ICIs did not affect CD8^+^ T cell migration even after adding CD4^+^ T cells (data not shown). Full representation of channels is available as **Supplement Figure 4D**.

**Figure 4:**
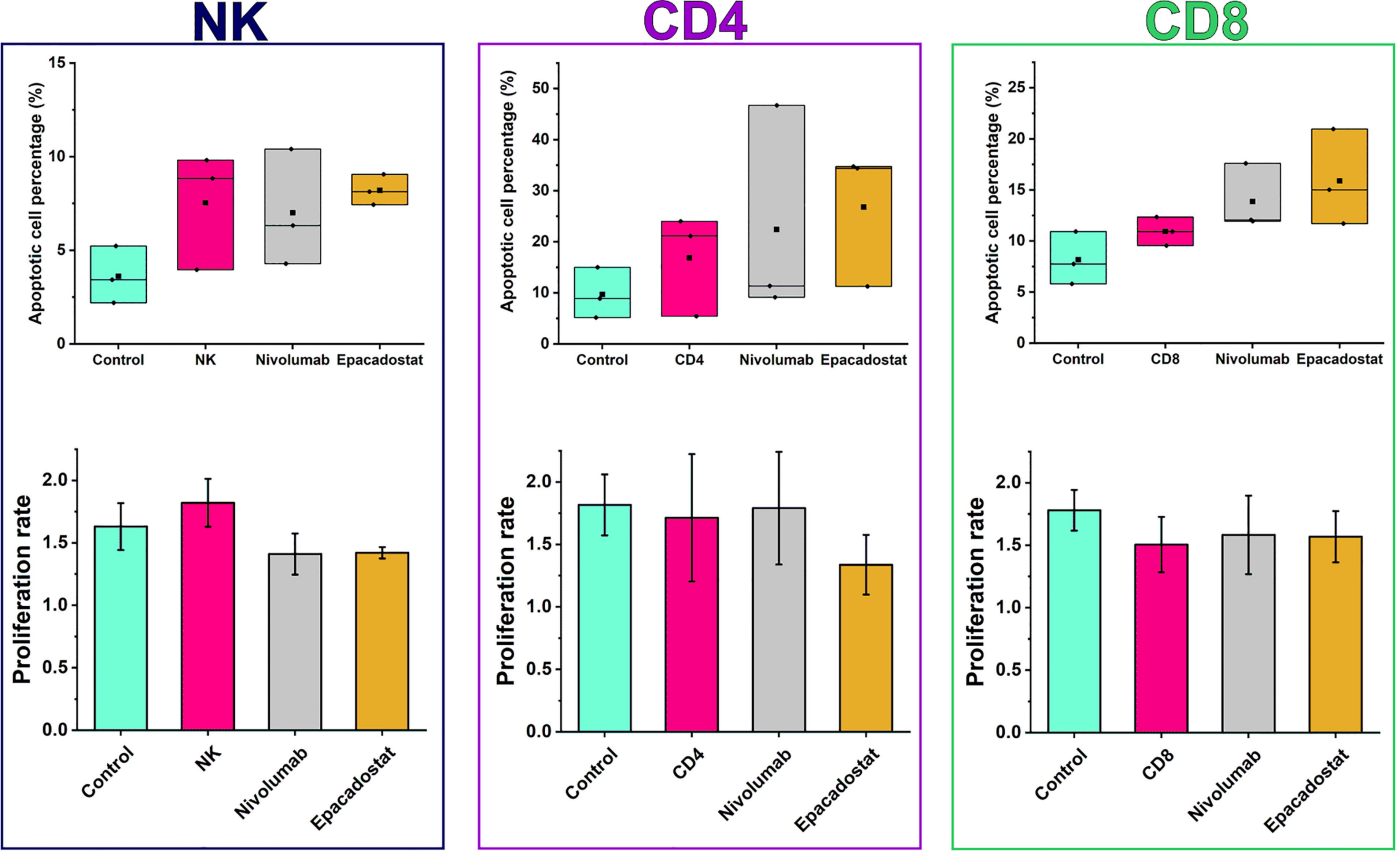
Apoptotic cell percentages and proliferation rates of HSC-3 in the presence or absence of lymphocytes with or without ICIs. There were no significant differences in apoptotic cell percentage or proliferation between the ICI groups and the lymphocyte or HSC-3 controls. Epacadostat treatment of NK cells exhibited a trend of increased apoptotic percentage and decreased proliferation rate than in the HSC-3 control but not in the NK cell control. Proliferation rates are reported as mean ± SD. The experiments were repeated three times, each time using a different donor and each in duplicate. *NK, natural killer cell; CD4, CD4^+^ T cells; CD8, CD8^+^ T cells*.

We next analyzed whether increased migration of lymphocytes affect cancer cell apoptosis and proliferation. There was a mild, but not significant, trend of increased apoptosis of HSC-3 cells after adding lymphocytes, especially in the presence of epacadostat (**Figure 4**). Proliferation was unaffected by lymphocytes alone or with either of the ICIs (**Figure 4**). Coculture of CD4^+^ T cells with CD8^+^ T cells did not affect apoptosis or proliferation of the cancer cells (data not shown).

### Nivolumab Significantly Increased MIP-1, IL-6, and IL-8 Release From NK, CD8^+^, and CD4^+^ T Cells, Respectively

**Figure 5:**
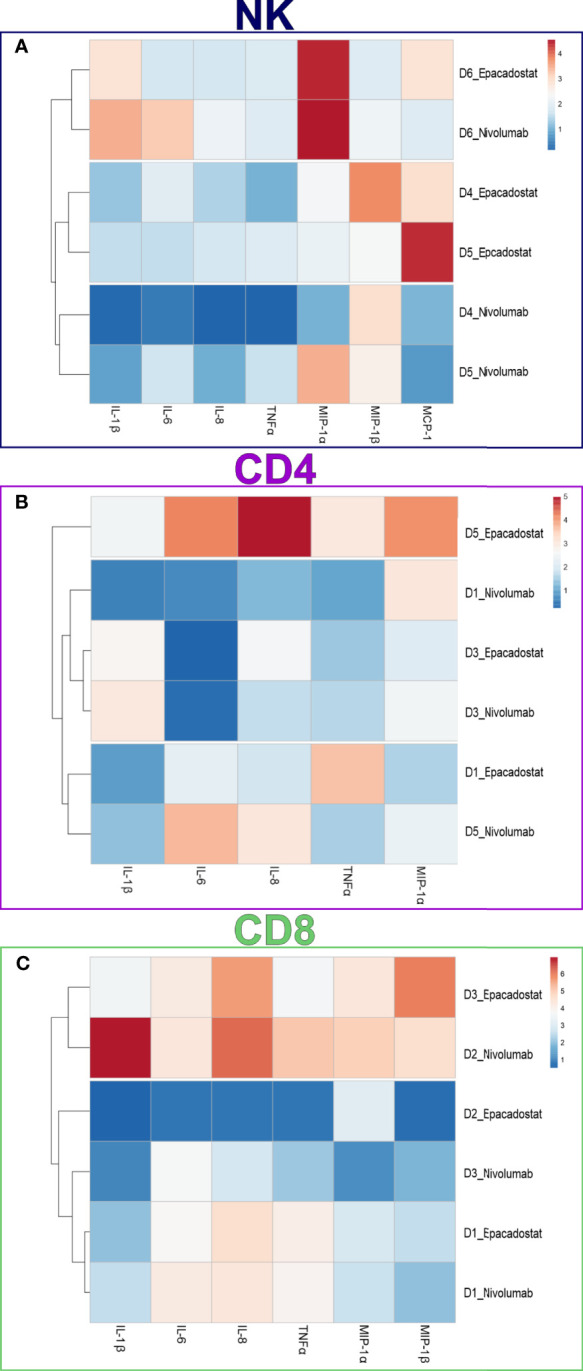
Cytokine expression in the conditioned media of NK, CD4^+^, and CD8^+^ T cells. Cytokine concentrations in the conditioned media were measured using a FirePlex^®^-96 Key Cytokines (Human) Immunoassay Panel. Cytokine concentrations were plotted in the heatmaps as fold change from control (HSC-3 alone). Heatmap of NK cells **(A)** showed apparent clustering for the ICIs epacadostat and nivolumab, with donor six showing donor variance. Heatmaps of the CD4^+^ T cells **(B)** and CD8^+^ T cells **(C)** did not show clustering based on ICI group. The experiments were repeated three times, each time using a different donor and each in duplicate. *NK, natural killer cell; CD4, CD4^+^ T cells; CD8, CD8^+^ T cells; D, donor; IL-1β, interleukin 1-beta; IL-6, interleukin 6; IL-8, interleukin 8; MCP-1, monocyte chemoattractant protein-1; MIP-1α, macrophage inflammatory protein 1 alpha; MIP-1β, macrophage inflammatory protein 1 beta; TNFα, tumor necrosis factor-alpha*.

We performed a FirePlex^®^-96 Key Cytokines (Human) Immunoassay Panel to study the effects of epacadostat and nivolumab on cytokine release of the lymphocytes in the presence of HSC-3 cells. Based on the cytokine fold change, samples collected from NK cell experiments clustered according to ICI stimulation except for donor 6, which clustered separately due to donor variation effect (**Figure 5A**). CD4^+^ and CD8^+^ T cells showed no clustering based on ICIs or donors (**Figures 5B, C**, respectively).

**Figure 6:**
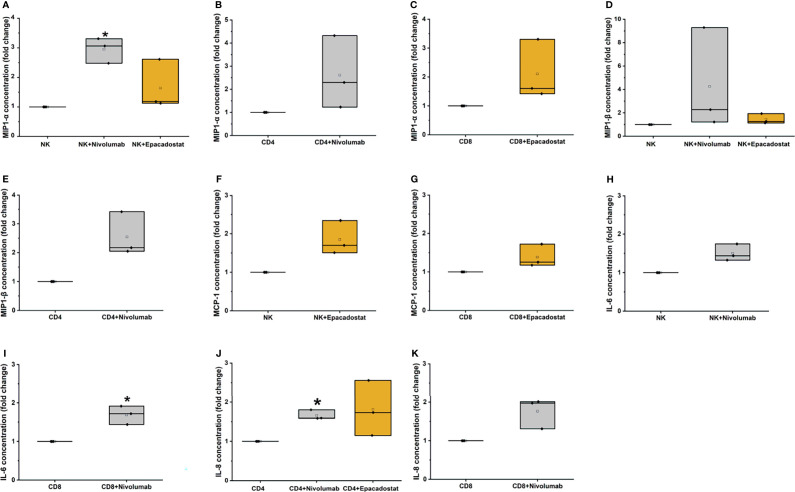
Cytokine analysis results for MIP-1α, MIP-1β, MCP-1, IL-6, and IL-8. MIP-1α levels increased significantly for NK cells with nivolumab treatment (**A**, *p=0.01). The CD4^+^ **(B)** and CD8^+^ T cells **(C)** showed a similar trend of increase with nivolumab and epacadostat treatment, respectively. MIP-1β levels exhibited an increasing trend for NK cells with nivolumab and epacadostat treatment **(D)**. CD4^+^ T cells had similarly elevated MIP-1β levels with nivolumab treatment **(E)**. MCP-1 levels were increased by CD8^+^ T **(F)** and NK cells **(G)** with epacadostat treatment. NK cells with nivolumab treatment exhibited an increasing trend in IL-6 levels **(H)**, while CD8^+^ T cells significantly elevated its levels (**I**, *p=0.038). IL-8 levels were significantly elevated for CD4^+^ T cells with nivolumab treatment (*p=0.011); epacadostat treatment also resulted in a similar increasing trend **(J)**. In addition, CD8^+^ T cells exhibited an increasing trend for IL-8 levels with nivolumab treatment **(K)**. Each dot represents one donor; the mean is presented as a small empty box and median as a black line. Grey box color indicates nivolumab and orange indicates epacadostat. The experiments were repeated three times, each time using a different donor and each in duplicate. *NK, natural killer cell; CD4, CD4^+^ T cells; CD8, CD8^+^ T cells; MIP-1α, macrophage inflammatory protein 1 alpha; MIP-1β, macrophage inflammatory protein 1 beta; MCP-1, monocyte chemoattractant protein-1; IL-6, interleukin 6; IL-8, interleukin 8*.

From the 17 cytokines studied, only after nivolumab treatment, the release of 3 cytokines MIP1-α from NK (p=0.016; **Figure 6A**), IL-6 from CD8^+^ T cells (p=0.038; **Figure 6I**), and IL-8 from CD4^+^ T cells (p=0.011; **Figure 6J**) were significantly increased. The other treatments exhibited similar trends but were not statistically significant (**Figure 6** and **Supplement Table 2**).

Incubation with epacadostat increased MIP1-α release from NK cells (**Figure 6A**). MIP1-α levels from CD4^+^ T cells treated with nivolumab and CD8^+^ T cells treated with epacadostat were increased (**Figures 6B, C**, respectively). With nivolumab treatment, NK and CD4^+^ T cells released increased MIP1-β (**Figures 6D, E**, respectively), which was also observed with epacadostat treatment of NK cells (**Figure 6D**). NK and CD8^+^ T cells incubated with epacadostat exhibited increased MCP-1 secretion (**Figures 6F, G**, respectively). Adding nivolumab to NK cells increased IL-6 concentration (**Figure 6H**). Although CD4^+^ T cells treated with epacadostat secreted increased IL-8 levels, the result was not significant (**Figure 6J**). Nivolumab treatment increased the amount of IL-8 released from CD8^+^ T cells (**Figure 6K**).

## Discussion

Despite the promising results of ICIs in some clinical trials, only approximately 13-18% of HNSCC patients respond to currently approved treatments (12, 21). Up to 60% of patients across different cancer types have primary resistance (21). The resistance mechanisms to ICI can be either tumor intrinsic or can develop during treatment. These include T cell exclusion or alterations in antigen presentation or cellular signaling pathways (22, 23). Unfortunately, there are currently no reliable predictive markers to identify patients who would benefit from ICIs, and the predictive role of PD-L1 expression levels and tumor-mutational burden remains controversial (20).

One proposed strategy to address resistance is to reverse T cell exclusion by stimulating trafficking and infiltration of leukocytes to “fire up cold tumors” (23). We have previously shown that IDO1 inhibition increased migration of PMNCs towards cancer cells (15). Here, to our knowledge, we are the first to report that IDO1 inhibition *via* epacadostat increased migration of NK and CD4^+^ T cells towards cancer cells. This could reverse the T cell exclusion in ICI-resistant “cold tumors” *in vivo*.

Epacadostat in combination with anti-PD1 therapy is being tested in several ongoing clinical trials. Combination therapy had promising results in phase 1 and 2 clinical trials for melanoma (24). However, its phase 3 clinical trial was cancelled in 2019 due to insufficient patient response, thus highlighting the need for additional in-depth preclinical studies before conducting large-scale, randomized clinical trials (24). We performed a preliminary test on a few microfluidic chips combining nivolumab and epacadostat but we did not observe any differences for any of the studied parameters (data not shown).

Despite epacadostat-induced lymphocyte migration, we did not observe a significant difference in apoptotic percentage or in proliferation rate of HSC-3 cells, which suggests a more complex underlying mechanism. In general, the ratio of cancer cells to leukocytes in the *in vitro* experiments ranged from 1:5 to 1:10, thus giving the leukocytes an advantage (13, 25, 26). In our experiment, we set the cancer cell-lymphocyte ratio at 1:100. However, the number of lymphocytes that actually migrated to the HSC-3 cells site was minimal and yielded a final ratio of approximately 7:1. To improve the accuracy of our model to capture even marginal changes, in the future we may consider decreasing the cancer cell to lymphocyte ratio in favor of lymphocytes. More viable lymphocytes and fewer cancer cells may yield realistic results. This would also prevent crowding of the cancer cells in the chip. Since the cancer cells were injected into a 3D environment, they grow on top of each other, which may cause an innate error to the visual analysis of the apoptotic cell layers.

Another proposed strategy to address ICI resistance are cytokine-based drugs (27). Cytokines are a group of potent but complex signaling molecules capable of modulating the immune response exerting both stimulatory and suppressive effects (27, 28). They mediate the expansion, activation and trafficking of effector lymphocytes but are also capable of recruiting regulatory T cells (27). In the TME, the lack of several cytokines, such as MIP1-β (CCL4), has been shown to lead to T-cell exclusion, while elevated IL-8 (CXCL8) expression has been shown to be associated with a reduction in the number of T cell in tumors (23). Various clinical trials are testing a combination therapy of cytokine-blockage and ICIs for different cancers (27). In addition to their potential therapeutic value, cytokines are also the molecules suggested to activate the effector lymphocytes after the ICI initiation (29). Several studies are evaluating the predictive value of inflammatory cytokine levels from patient serum before and after ICI therapy in different cancers (29–32).

Cytokine levels from the conditioned media were analyzed to explore the effect of ICIs on the signaling pathways that underlie the cross-talk between lymphocytes and HNSCC cells. With the exception of one donor, samples from nivolumab and epacadostat clustered differently for NK cells. Donor variation was further confirmed, as donor 3 exhibited high concentrations of nearly all cytokines, suggesting that this donor had underlying immune system activation due to inflammatory disease.

Both nivolumab and epacadostat had mild effects on cytokine levels, but only nivolumab had some statistically significant results. TNF-α, MIP1-α, MIP1-β, MCP-1, IL-6, and IL-8 had the highest cytokine levels for all donors. The TNF-α concentration varied without any trends between groups. The role of the proinflammatory chemokines MIP1-α (CCL3), MIP1-β (CCL4), and MCP-1 (CCL2) in the tumor remains controversial, as described below.

MIP1-α mRNA expression is increased in oral squamous cell carcinoma (OSCC) when compared with healthy gingival tissue (33). Furthermore, MIP1-α serum levels were suggested as a potential biomarker for diagnosing OSCC, as these levels are associated with tumor size (34). We observed a significant increase in MIP1-α levels for NK cells after nivolumab treatment (similar to epacadostat), but this was not significant. CCR5 (a receptor for MIP1-α) activation increases migration of regulatory T cells, thus promoting immune evasion. Interestingly, CCR5 blockade in hepatocellular carcinoma had promising results with maraviroc, which was initially developed as a human immunodeficiency virus medication (#NCT01736813; 34). At present, there are three ongoing studies investigating combinations of pembrolizumab and maraviroc for metastatic carcinomas (35).

Plasma levels of MIP1-β were downregulated in OSCC patients compared to the healthy controls. High expression of MIP1-β is linked to anti-tumor responses through chemoattraction of lymphocytes (T and NK cells) in esophageal SCC and colorectal adenocarcinomas (36–38). Here, no significant results were obtained for MIP1-β levels. However, epacadostat treatment showed a trend of increased of MIP1-β secretion from NK cells and nivolumab treatment showed a trend of increased MIP1-β secretion from NK and CD4^+^ T cells. On the other hand, MIP1-β is overexpressed in lung adenocarcinomas and colorectal carcinomas, which is linked to tumor development and progression through protumorigenic macrophage recruitment (39, 40). Moreover, a case report of an OSCC patient showed elevated level of MIP1-β secretion but with no response to nivolumab treatment. This suggests that the functional mechanisms of MIP1-β require further studies (41).

MCP-1 is a potent monocyte-attracting chemokine that improves monocyte recruitment to the TME and promotes HNSCC progression (42). Moreover, OSCC-associated fibroblasts mediate protumorigenic features through MCP-1 signaling (43). On the other hand, *in vivo* MCP-1 elicits effector T cell chemotaxis (44), but its role in recruiting T cells to the TME is still unclear (45). In our study, NK and CD8^+^ T cells showed a trend of elevated MCP-1 levels with epacadostat treatment.

We demonstrated that IL-6 and IL-8 (CXCL8) levels were significantly elevated after nivolumab treatment of CD8^+^ and CD4^+^ T cells compared with controls. In various cancers, the elevated systemic and tumor-associated levels of IL-6 and IL-8 are associated with reduced clinical benefit of anti-PD-1/PD-L1 treatment (46, 47). Furthermore, increased IL-6 and IL-8 serum levels after anti-PD-1/PD-L1 treatment are associated with no response in non-small cell lung carcinoma and melanoma patients (30–32). In OSCC patients, IL-6 and IL-8 serum levels are elevated (48–50). However, to our knowledge, there is only one case report where some cytokines before and after treatment were analyzed (41). Similar to our results, this patient had elevated IL-6 and IL-8 levels after nivolumab treatment, which was associated with disease progression (41). To our knowledge, we are the first to report the actual lymphocytes mediating the increased levels of IL-6 and IL-8. Elevated IL-6 levels compared to the respective lymphocyte control were observed for CD8^+^ T cells with nivolumab treatment, while NK cells showed a similar trend. Increased IL-8 levels were observed for CD4^+^ T cells with nivolumab treatment, while epacadostat incubation showed a similar trend. Additionally, nivolumab treatment also increased IL-8 levels from CD8^+^ T cells. Blockage of IL-6 and IL-8 in combination with nivolumab is being investigated in phase 1 clinical trials for several carcinomas, including HNSCC for IL-8 (clinical trial identifiers #NCT03400332, #NCT04848116, #NCT03999749).

Due to the limited availability of blood samples and carcinoma cells from HNSCC patients, we used lymphocytes harvested from healthy individuals and a commercial OSCC cell line. Although this was not ideal, obtaining sufficient lymphocytes and cancer cells from the same patient is not feasible. Therefore, methodologies similar to ours, where leukocytes are isolated from healthy individuals, have commonly been used in the literature (25, 26, 51). As our results show variable effects depending on ICI on OSCC cell migration and apoptosis and on lymphocyte cytokine secretion, further *in vivo* experiments are warranted to validate these findings.

## Conclusions

We showed that epacadostat stimulated migration of NK and CD4^+^ T cells towards the site of carcinoma cells, thus potentially enhancing antigen presentation in the TME. Nivolumab did not affect cell migration. However, in the presence of nivolumab, NK, CD4^+^, and CD8^+^ T cells secreted more MIP1-α, IL-6, and IL-8, respectively, than controls. Importantly, since increased levels of IL-6 and IL-8 in serum from OSCC patients after anti-PD1 treatment are associated with poor ICI response, blockage of these cytokines may be a potential target for clinical trials. Moreover, levels of these cytokines could potentially reflect patient response to anti-PD1 treatment, which should be further investigated. Blockage of the MIP1-α receptor may also be a promising direction for future studies.

## Data Availability Statement

The original contributions presented in the study are included in the article/**Supplementary Material**. Further inquiries can be directed to the corresponding author.

## Ethics Statement

The studies involving human participants were reviewed and approved by the ethical committee of the Finnish Red Cross (permission number 42/2020). Written informed consent for participation was not required for this study in accordance with the national legislation and the institutional requirements.

## Author Contributions

MS designed and performed experiments, analyzed the results, and wrote the manuscript. JS performed the experiments, imaged the chips, and analyzed the results. SA-A extracted the lymphocytes and analyzed the results. TS supervised the project, designed the experiments, and interpreted the results. AA supervised the project, designed the experiments, and interpreted the results. All authors edited and approved the manuscript.

## Funding

MS was financially supported by a fellowship from the Doctoral Programme in Oral Sciences. The project was funded by the Niilo Helander Foundation, the Maja och Lisa Selander Foundation, Suomen Naishammaslääkäriseura, the Cancer Society of Finland, the Sigrid Juselius Foundation, the Finnish Dental Society Apollonia, the Jane and Aatos Erkko Foundation, and Helsinki University Central hospital research funds. The microfluidic solutions and devices were provided by Probiont Oy, Helsinki, Finland as direct support to this study.

## Conflict of Interest

The authors declare that the research was conducted in the absence of any commercial or financial relationships that could be construed as a potential conflict of interest.

## Publisher’s Note

All claims expressed in this article are solely those of the authors and do not necessarily represent those of their affiliated organizations, or those of the publisher, the editors and the reviewers. Any product that may be evaluated in this article, or claim that may be made by its manufacturer, is not guaranteed or endorsed by the publisher.

## Supplementary Material

Supplementary Figure 1 Purity of natural killer **(A)**, CD4^+^ T **(B)**, and CD8^+^ T cells **(C)** acquired using flow cytometer FACS-Verse. Natural killer cells were stained with anti-CD3 APC, anti-CD56 PE Cy7, and anti-CD4 PE. CD4^+^ and CD8^+^ T cells were stained for anti-CD3 APC, anti-CD4 PE, and anti-CD8 FITC.

Supplementary Figure 2 Neither nivolumab nor epacadostat affects HSC-3 viability. Four different concentrations of nivolumab and epacadostat were screened for their cytotoxic effect on HSC-3 cells using a CellTiter-Glo assay. Viability did not significantly (p>0.05) vary between any tested concentrations of nivolumab **(A)** or epacadostat **(B)**. Results are presented as means normalized to control wells without drugs ± SD. The assays were performed as duplicates and repeated three times independently.

Supplementary Figure 3 Semi-automated analysis of coculture chips. Coculture chips of CD4^+^ and CD8^+^ T cells were imaged under four fluorescent filters to obtain a multichannel image **(A)**, with HSC-3 cells in the red channel, CD4^+^ T cells in the blue channel, apoptotic cells in the green channel, and CD8^+^ T cells in the orange channel. The number of cancer cells, apoptotic cancer cells, and CD4^+^ T cells were calculated as in the three-dye system. For analysis of the number of CD8^+^ T cells, the green channel containing apoptotic cells was removed **(B)** and the orange channel containing the CD8^+^ T cells was changed to blue and overlayed with the red channel only **(C)**. The software then calculated all positive blue cells with red intensity **(D)** and all positive blue cells with the same parameters as the CD4^+^ T cells in the three-dye system **(E)**. Subtraction of these cells was considered to yield the number of CD8^+^ T cells. To provide a clear representation of the migrated cells, the representative image of the cocultures was edited such that the cancer cells are shown in white, CD4^+^ T cells in blue, and CD8^+^ T cells in magenta (F). Scale bar A-E 100 µm.

Supplementary Figure 4 Full representation of analyzed channels on day 3 for NK cells, CD4^+^ T cells, CD8^+^ T cells, and CD4^+^ T cells with CD8^+^ T cells. In the monoculture chip images **(A–C)**, HSC-3 cells are shown in red, respective lymphocytes (NK cells, CD4^+^ T cells, and CD8^+^ T cells) in blue, and apoptotic cells in green. Apoptotic cancer cells appeared yellow due to overlay of red and green colors. In coculture chip images **(D)**, HSC-3 cells are shown in grey, CD4^+^ T cells in blue, CD8^+^ T cells in magenta, and apoptotic cells in green. Scale bar A-D 300 µm.

Supplementary Table 1 Clinical characteristics of the six donors.

Supplementary Table 2 Cytokine concentrations of the conditioned media. Values are in pg/ml. D, donor.
