# Supplementary figures and images for: IDO1 Inhibition Reduces Immune Cell Exclusion Through Inducing Cell Migration While PD-1 Blockage Increases IL-6 and -8 Secretion From T Cells in Head and Neck Cancer
Source: Front Immunol. 2022 Mar 14;13:812822. doi: 10.3389/fimmu.2022.812822 (PMC8963946; doi:10.3389/fimmu.2022.812822)

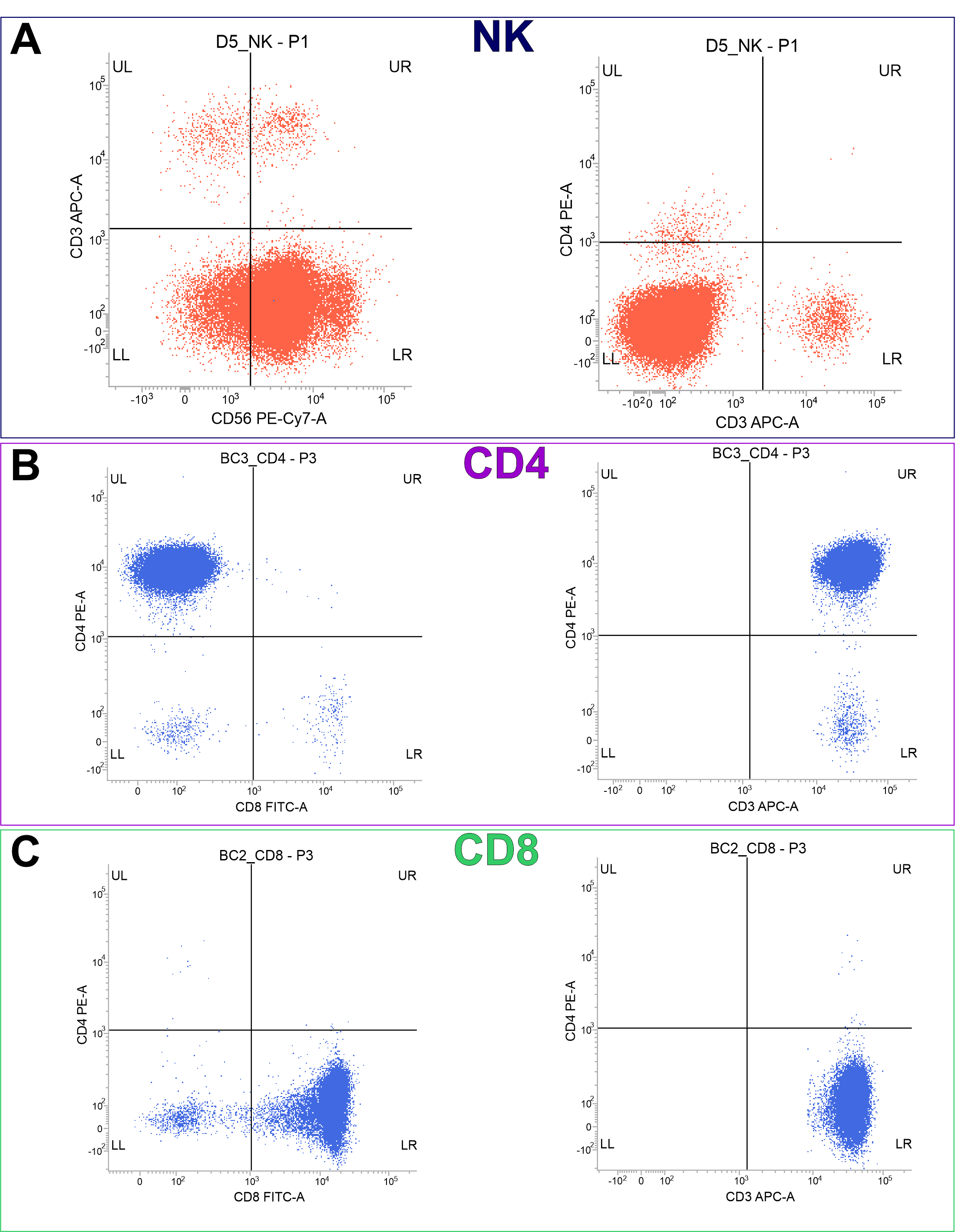

Supplement: Supplementary Figure 1 — Purity of natural killer (A), CD4+ T (B), and CD8+ T cells (C) acquired using flow cytometer FACS-Verse. Natural killer cells were stained with anti-CD3 APC, anti-CD56 PE Cy7, and anti-CD4 PE. CD4+ and CD8+ T cells were stained for anti-CD3 APC, anti-CD4 PE, and anti-CD8 FITC. [file Image_1.tif]

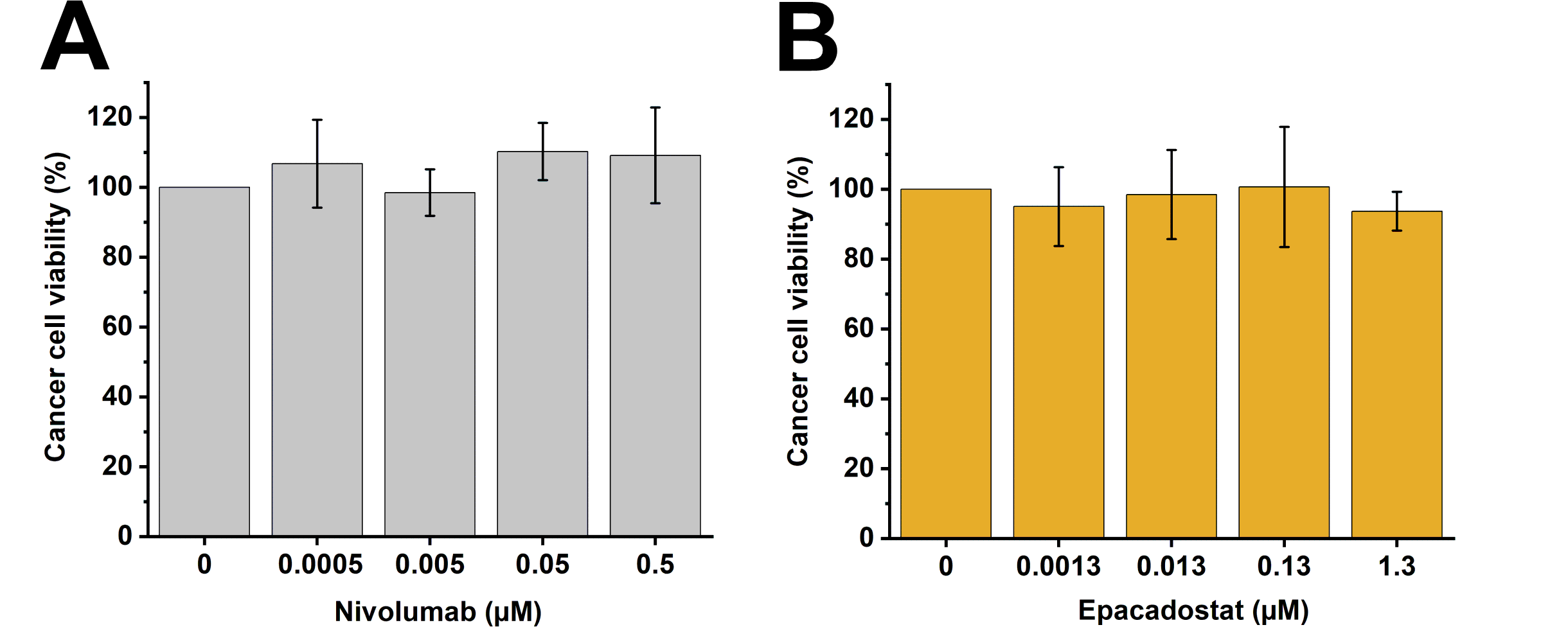

Supplement: Supplementary Figure 2 — Neither nivolumab nor epacadostat affects HSC-3 viability. Four different concentrations of nivolumab and epacadostat were screened for their cytotoxic effect on HSC-3 cells using a CellTiter-Glo assay. Viability did not significantly (p>0.05) vary between any tested concentrations of nivolumab (A) or epacadostat (B). Results are presented as means normalized to control wells without drugs ± SD. The assays were performed as duplicates and repeated three times independently. [file Image_2.tif]

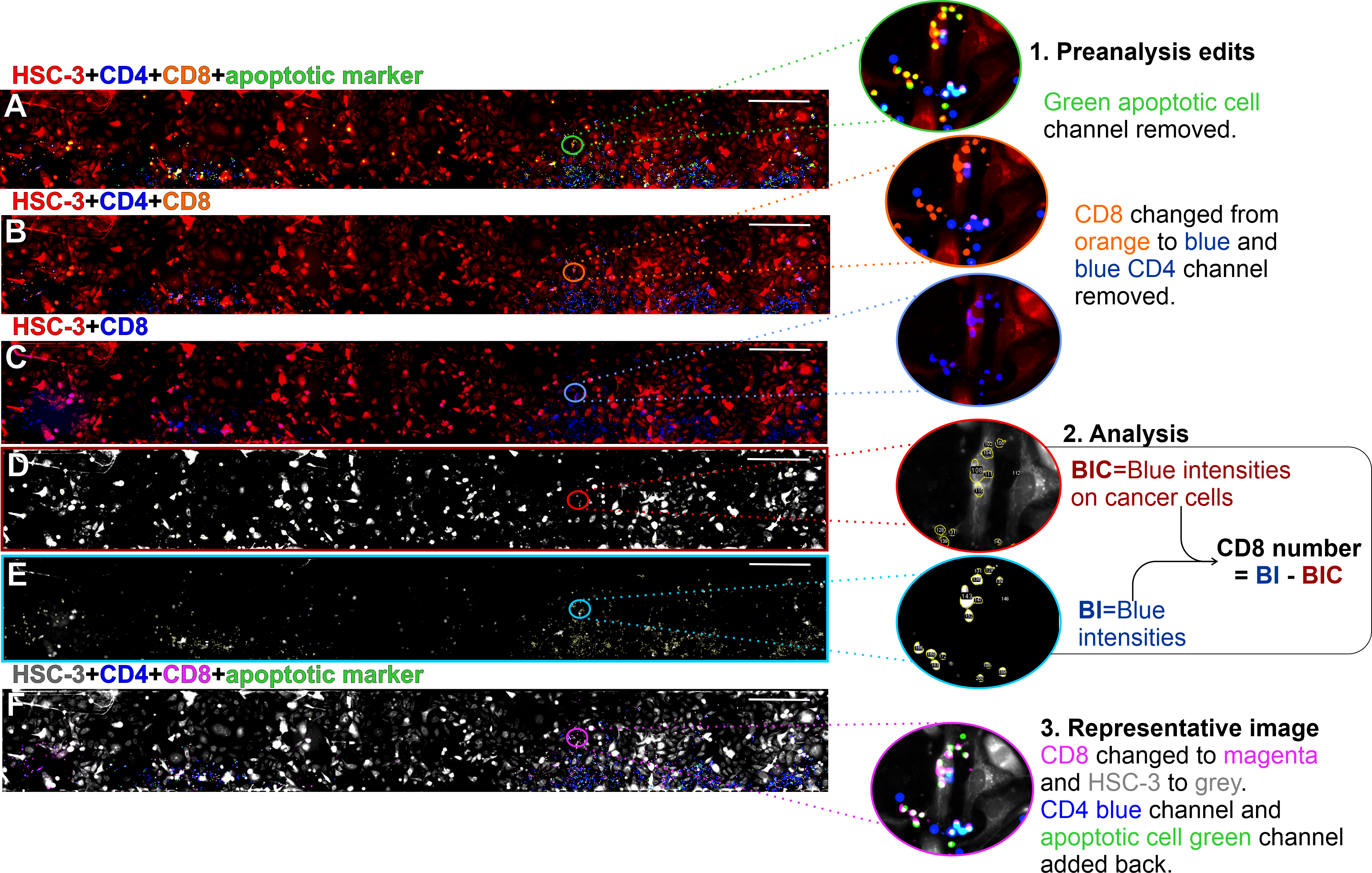

Supplement: Supplementary Figure 3 — Semi-automated analysis of coculture chips. Coculture chips of CD4+ and CD8+ T cells were imaged under four fluorescent filters to obtain a multichannel image (A), with HSC-3 cells in the red channel, CD4+ T cells in the blue channel, apoptotic cells in the green channel, and CD8+ T cells in the orange channel. The number of cancer cells, apoptotic cancer cells, and CD4+ T cells were calculated as in the three-dye system. For analysis of the number of CD8+ T cells, the green channel containing apoptotic cells was removed (B) and the orange channel containing the CD8+ T cells was changed to blue and overlayed with the red channel only (C). The software then calculated all positive blue cells with red intensity (D) and all positive blue cells with the same parameters as the CD4+ T cells in the three-dye system (E). Subtraction of these cells was considered to yield the number of CD8+ T cells. To provide a clear representation of the migrated cells, the representative image of the cocultures was edited such that the cancer cells are shown in white, CD4+ T cells in blue, and CD8+ T cells in magenta (F). Scale bar A-E 100 µm. [file Image_3.tif]

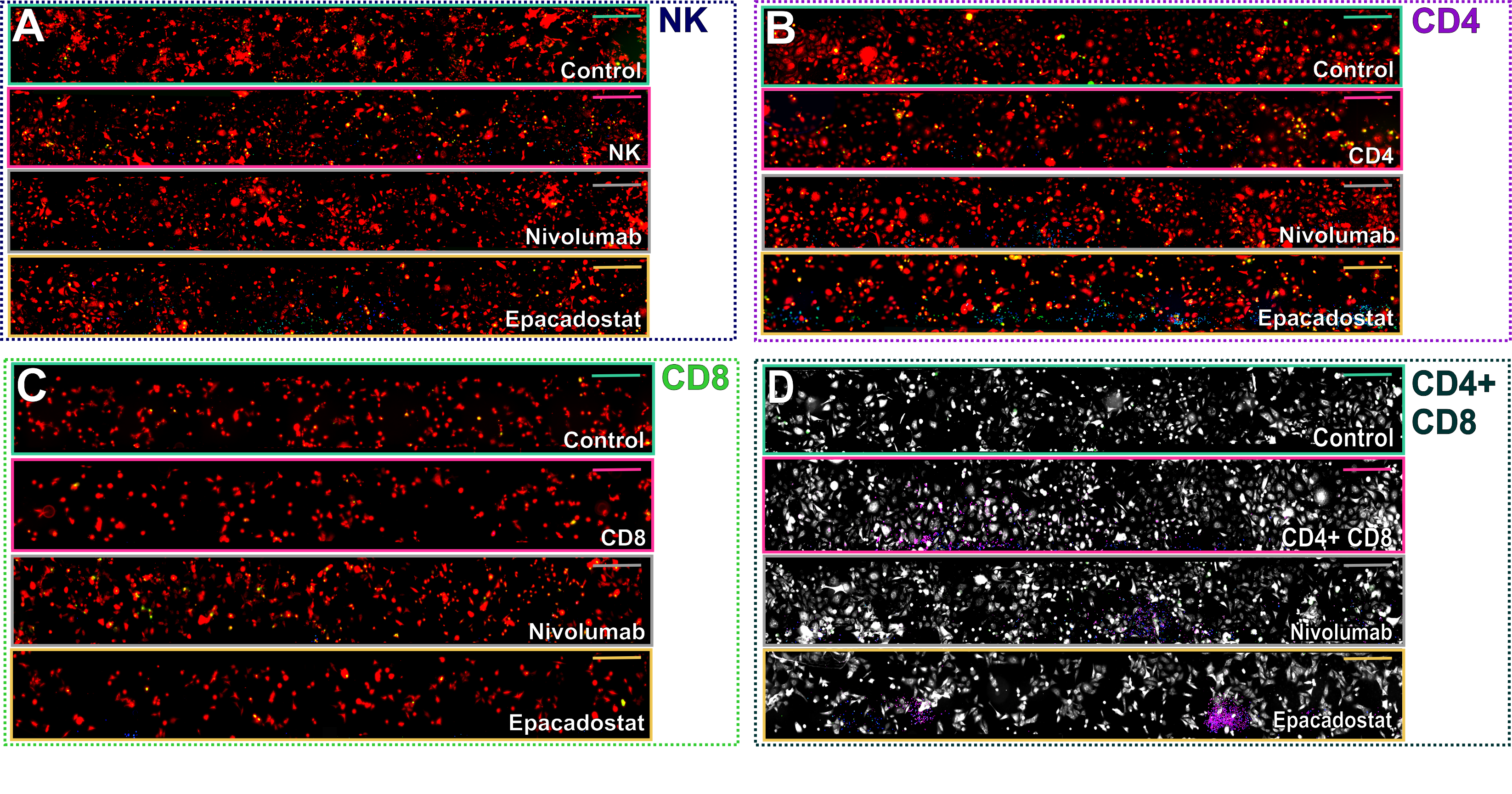

Supplement: Supplementary Figure 4 — Full representation of analyzed channels on day 3 for NK cells, CD4+ T cells, CD8+ T cells, and CD4+ T cells with CD8+ T cells. In the monoculture chip images (A–C), HSC-3 cells are shown in red, respective lymphocytes (NK cells, CD4+ T cells, and CD8+ T cells) in blue, and apoptotic cells in green. Apoptotic cancer cells appeared yellow due to overlay of red and green colors. In coculture chip images (D), HSC-3 cells are shown in grey, CD4+ T cells in blue, CD8+ T cells in magenta, and apoptotic cells in green. Scale bar A-D 300 µm. [file Image_4.tif]
