# Supplementary material for: IDO1 Inhibition Reduces Immune Cell Exclusion Through Inducing Cell Migration While PD-1 Blockage Increases IL-6 and -8 Secretion From T Cells in Head and Neck Cancer
Source: Front Immunol. 2022 Mar 14;13:812822. doi: 10.3389/fimmu.2022.812822 (PMC8963946; doi:10.3389/fimmu.2022.812822)
Supplement: Supplementary Table 1 — Clinical characteristics of the six donors. [file Table_1.docx]

| No | Lymphocytes extracted | Age | Gender |
| --- | --- | --- | --- |
| Donor 1 | CD4, CD8 | 63 | Male |
| Donor 2 | CD8 | 28 | Female |
| Donor 3 | CD4, CD8 | 61 | Male |
| Donor 4 | CD4, NK | 46 | Female |
| Donor 5 | NK | 60 | Female |
| Donor 6 | CD4, CD8, NK | 46 | Male |

*No=number; CD4=CD4^+^ T cells; CD8=CD8^+^ T cells; NK=Natural Killer Cells*
