# Supplementary material for: IDO1 Inhibition Reduces Immune Cell Exclusion Through Inducing Cell Migration While PD-1 Blockage Increases IL-6 and -8 Secretion From T Cells in Head and Neck Cancer
Source: Front Immunol. 2022 Mar 14;13:812822. doi: 10.3389/fimmu.2022.812822 (PMC8963946; doi:10.3389/fimmu.2022.812822)
Supplement: Supplementary Table 2 — Cytokine concentrations of the conditioned media. Values are in pg/ml. D, donor. [file Table_2.docx]

| **CYTOKINE CONCENTRATIONS OF THE CONDITIONED MEDIA.**  **VALUES ARE IN pg/ml. D: DONOR** | | | | |
| --- | --- | --- | --- | --- |
| **IL-1β** | **Control (HSC-3)** | **Lymphocyte Control (HSC-3+lymphocyte)** | **Epacadostat** | **Nivolumab** |
| NK D4 | 0,110263859 | 0,179922745 | 0,1141421 | 0,140140098 |
| NK D5 | 0,11853051 | 0,202964986 | 0,16903326 | 0,153347471 |
| NK D6 | 0,127915736 | 0,123293857 | 0,20928349 | 0,233281434 |
| CD4 D1 | 0,083050531 | 0,192613969 | 0,24737746 | 0,164657504 |
| CD4 D3 | 0,143246089 | 1,704056282 | 2,06213765 | 4,661916425 |
| CD4 D4 | 0,156582945 | 0,186923652 | 0,16261789 | 0,133230354 |
| CD8 D1 | N/A | 0,504379045 | 0,28822557 | 1,152169259 |
| CD8 D2 | 0,253810421 | 0,200121155 | 0,22730226 | 0,232187313 |
| CD8 D3 | 0,261939267 | 0,625567624 | 0,74031336 | 0,322419855 |
| **IL-6** | **Control (HSC-3)** | **Lymphocyte Control (HSC-3+lymphocyte)** | **Epacadostat** | **Nivolumab** |
| NK D4 | 2,097866984 | 2,798788639 | 2,79878864 | 3,705585344 |
| NK D5 | 1,15553949 | 3,861292863 | 3,24238461 | 5,549797491 |
| NK D6 | 1,211635076 | 0,835706173 | 0,84776714 | 1,458829048 |
| CD4 D1 | 1,473313228 | 1,659737389 | 5,1464041 | 1,690910885 |
| CD4 D3 | 2,086279131 | 4,366404053 | 0,52381818 | 1,534908499 |
| CD4 D4 | 1,494014943 | 0,908396236 | 1,33081806 | 1,762966324 |
| CD8 D1 | 1,15553949 | 3,861292863 | 3,24238461 | 5,549797491 |
| CD8 D2 | 6,22337316 | 1,231988902 | 2,60222796 | 2,357995711 |
| CD8 D3 | 0,157571824 | 2,227820431 | 3,10878481 | 3,830701908 |
| **IL-8** | **Control (HSC-3)** | **Lymphocyte Control (HSC-3+lymphocyte)** | **Epacadostat** | **Nivolumab** |
| NK D4 | 63,65399525 | 82,86313152 | 60,4685913 | 49,32385958 |
| NK D5 | 78,25236909 | 111,2132056 | 103,432188 | 99,15259491 |
| NK D6 | 74,42105202 | 70,12124947 | 71,1303329 | 82,84881099 |
| CD4 D1 | 20,03332707 | 37,2336466 | 95,2503049 | 67,15014567 |
| CD4 D3 | 14,94079225 | 1879,337812 | 2161,06103 | 2992,106341 |
| CD4 D4 | 20,03332707 | 33,67496401 | 58,4220806 | 53,43688608 |
| CD8 D1 | 12,26823815 | 111,2132056 | 94,7720453 | 223,9731914 |
| CD8 D2 | 32,38966518 | 21,11681861 | 54,5248822 | 41,66899088 |
| CD8 D3 | 7,3276363 | 57,67429398 | 107,013825 | 75,5139222 |
| **MCP-1** | **Control (HSC-3)** | **Lymphocyte Control (HSC-3+lymphocyte)** | **Epacadostat** | **Nivolumab** |
| NK D4 | NA | 0,526686018 | 0,7940332 | 1,820028576 |
| NK D5 | NA | 7,464997258 | 17,4896941 | 5,198602259 |
| NK D6 | 1,367194525 | 1,466592647 | 2,49181646 | 1,510702513 |
| CD4 D1 | NA | NA | 0,27918866 | 0,21245353 |
| CD4 D3 | 0,318128715 | 11,33799536 | 0,02415613 | 5,753845558 |
| CD4 D4 | 0,473017248 | NA | 0,53389266 | 0,507487055 |
| CD8 D1 | NA | 0,311883328 | 0,39083811 | 0,929751475 |
| CD8 D2 | NA | 0,317075451 | 0,37265634 | 0,294837308 |
| CD8 D3 | NA | 0,271479558 | 0,46746412 | NA |
| **MIP-1α** | **Control (HSC-3)** | **Lymphocyte Control (HSC-3+lymphocyte)** | **Epacadostat** | **Nivolumab** |
| NK D4 | 0,071492324 | 0,41643842 | 0,4923276 | 1,37833901 |
| NK D5 | 0,02758253 | 7,130043082 | 8,03116448 | 21,85291168 |
| NK D6 | 0,620791014 | 0,510068926 | 1,33312695 | 1,263567874 |
| CD4 D1 | 0,024670834 | 0,938418508 | 2,05827356 | 4,053407786 |
| CD4 D3 | 0,025076491 | 84,89240252 | 80,39607 | 194,6748518 |
| CD4 D4 | NA | 4,246176677 | 6,098098 | 5,225381888 |
| CD8 D1 | 0,022973264 | 13,53264583 | 44,7168694 | 21,91042472 |
| CD8 D2 | 0,027629346 | 0,635445205 | 1,01976809 | 0,757707339 |
| CD8 D3 | 0,051677188 | 6,17012368 | 8,76352295 | 3,45874745 |
| **MIP-1β** | **Control (HSC-3)** | **Lymphocyte Control (HSC-3+lymphocyte)** | **Epacadostat** | **Nivolumab** |
| NK D4 | NA | 0,038065205 | 0,0735558 | 0,353626261 |
| NK D5 | NA | 0,758980856 | 0,9520097 | 1,725317103 |
| NK D6 | 0,454325865 | 0,579348952 | 0,65785809 | 0,705508014 |
| CD4 D1 | NA | 0,229655095 | 0,39085359 | 0,78443087 |
| CD4 D3 | 0,024156133 | 20,88408148 | 18,4012839 | 45,34339123 |
| CD4 D4 | NA | 1,106724694 | 1,70035149 | 2,267586543 |
| CD8 D1 | NA | 6,783124805 | 4,77868121 | 10,23704203 |
| CD8 D2 | NA | 0,729371598 | 1,04673451 | 0,667675301 |
| CD8 D3 | NA | 0,893337517 | 1,74107444 | 0,76575201 |
| **TNF-α** | **Control (HSC-3)** | **Lymphocyte Control (HSC-3+lymphocyte)** | **Epacadostat** | **Nivolumab** |
| NK D4 | 1,019776628 | 2,249394144 | 1,18365084 | 1,324446579 |
| NK D5 | 1,03796207 | 1,182604229 | 1,189804 | 1,679688866 |
| NK D6 | 0,736465653 | 0,805272127 | 0,88812116 | 0,845985098 |
| CD4 D1 | 0,24728079 | 0,867794506 | 4,2714997 | 1,283328411 |
| CD4 D3 | 0,349099782 | 28,73334957 | 18,6709077 | 43,70606384 |
| CD4 D4 | 0,841151652 | 1,743457695 | 1,83044768 | 1,418923369 |
| CD8 D1 | 0,237872279 | 1,609493924 | 1,35335714 | 2,677118426 |
| CD8 D2 | 0,445984498 | 0,616337525 | 1,42032626 | 1,090694033 |
| CD8 D3 | 0,274035453 | 1,164788679 | 1,40087738 | 1,161450345 |
